# Carcass Composition and Quality of Meat of Pulawska and Pulawska x PLW Crossbred Pigs Fed Rations with Naked Oats

**DOI:** 10.3390/ani11123342

**Published:** 2021-11-23

**Authors:** Anna Milczarek

**Affiliations:** Institute of Animal Science and Fisheries, Faculty of Agrobioengineering and Animal Husbandry, Siedlce University of Natural Sciences and Humanities, Bolesława Prusa 14, 08-110 Siedlce, Poland; anna.milczarek@uph.edu.pl; Tel.: +48-25-643-13-77

**Keywords:** naked oats, performance results, carcass composition, physical-chemical properties of meat, fattening pigs

## Abstract

**Simple Summary:**

Due to the presence of fibrous hulls, the use of traditional cultivars of oats (*Avena sativa* L.) in feed rations for pigs is restricted. The development of naked oat cultivars *(Avena nuda* L.) containing only about 2% of crude fibre allowed this cereal grain to be included in the diets of monogastric animals. The present studies evaluated the efficiency of a 60% inclusion of naked oats in rations fed to Pulawska pigs and Pulawska x Polish Large White (PLW) crossbreds on their performance results (weight gain, feed conversion), carcass composition and physical (pH, colour, WHC) and chemical (essential nutrients, fatty acids) characteristics of meat. It was demonstrated that a 60% inclusion of naked oats is recommended in the diets of Pulawska pigs and Pulawska x PLW crossbreds to maintain carcass composition and meat quality comparable to that of pigs fed with rations containing barley only. The performance indicators of Pulawska pigs declined, but their meat quality traits improved in terms of nutritional value, as the n-6/n-3 acids’ ratio was narrower than that of Pulawska x PLW.

**Abstract:**

The study aimed to determine the impact of a 60% inclusion of naked oats in feed rations for Pulawska pigs and Pulawska x Polish Large White (PLW) crossbreds on the fattening performance, carcass composition, and meat quality. It was demonstrated that—independent from their diet—Pulawska pigs showed a daily weight gain about 14.5% lower (*p* ≤ 0.05), with about 15% higher feed conversion rate. The experimental factors showed no impact on the dressing percentage, meatiness, and backfat thickness, except that the “eye” of the loin was significantly smaller (by 4.55 cm^2^) in Pulawska pigs. There was no interaction effect (diet/breed) for the pigs’ performance results and carcass composition. A 60% inclusion of naked oats in the pigs’ diet did not affect the weight of primal cuts in the right-side half-carcass. Compared to Pulawska pigs, crossbreds featured a significantly lower weight of bacon and ribs (by 1.17 kg) but a higher (*p* ≤ 0.05) weight of fillet (by 1.0 kg) and ham with shin (by 0.43 kg). The diet had no impact on the evaluated muscles, except a reduction (by 2.3 points) in colour lightness (L) of *musculus semimembranosus* in fattening pigs receiving feed rations with a 60% share of oats. Both muscles in Pulawska pigs showed significantly better (*p* ≤ 0.05) water-holding capacity and reduced colour lightness (L) in comparison to crossbred pigs. Moreover, the *longissimus lumborum* muscle of Pulawska pigs had a higher chroma (C) and a lower hue (H). The contents of essential nutrients in the evaluated muscles did not depend on the pigs’ diet and breed, except that a higher by 0.3% (*p* ≤ 0.05) intramuscular fat content was found in the *longissimus lumborum* muscle of Pulawska pigs. Neither of the experimental factors showed significant impact on the total saturated fatty acids (SFA) and total unsaturated fatty acids (UFA), or on neutral or hypocholesterolemic (DFA) and hypercholesterolemic fatty acids (OFA) in the evaluated muscles. The muscles of Pulawska x PLW pigs contained more (by 1.77% FA in the *longissimus lumborum* and 1.16% in the *semimembranosus*) polyunsaturated fatty acids (PUFA) than the muscles of the Pulawska breed (*p* ≤ 0.05). In addition, naked oats included in the pigs’ diet increased (*p* ≤ 0.05) the share of PUFA in intramuscular fat. The muscles of Pulawska pigs, in comparison to the muscles of crossbreds, showed a significantly improved ratio: by 66% and 69% in the *longissimus lumborum* and the *semimembranosus* muscles, respectively. To sum up, a 60% inclusion of naked oats is recommended in the rations of both Pulawska pigs and Pulawska x PLW crossbreds, since it allows satisfactory carcass composition and meat quality to be maintained. Pulawska pigs had worse productivity ratios but showed improved meat quality traits in terms of the n-6/n-3 ratio.

## 1. Introduction

Pork accounts for nearly 40% of the global production and consumption of meat [1]. It is a source of complete protein and significant amounts of easily assimilated iron, zinc, selenium, copper, and vitamins from the B group, as well as bioactive compounds [2,3,4]. The content of fat and its fatty acid profile are nutritionally essential [5,6]. Increased awareness makes consumers look for culinary meat of high quality, reflected in sensory traits and health properties. Most consumers prefer light pink meat with minimum fat and juice drip. A darker colour of meat and excessive drip are associated with the loss of freshness [7]. For heat-treated meat, the main consumer focus is on its sensory quality, which is dependent mostly on juice drip and fat content. All the above-mentioned characteristics affect the quality of the meat, which depend on the breed of pigs, their genotype, pigs’ diet, and handling of animals before and after slaughter [3,8,9,10]. Pork from Pulawska pigs can be an attractive raw material for high-quality meat products. The Pulawska is a native breed from the eastern Lublin region and western Masovia in Poland. Compared with typical meat pig breeds, it features lower fattening ratios (lower daily weight gain and increased feed conversion), lower meatiness, and slightly increased fat percentage, but the meat is clearly of higher quality [2,10,11,12,13]. A significant element contributing to the high quality of pork meat from Pulawska pigs is the structure of the muscle fibres and the content of intramuscular fat (IMF), determining its palatability. As reported by Kasprzyk [11], Babicz [14], and Wojtysiak and Połtowicz [15], this creates a possibility of using Pulawska pigs for producing brand-name products, e.g., Pulawska ham. Bocian et al. [16], Debrecéni et al. [17], Matoušek et al. [18], and Colonna et al. [19] claimed that native breeds are raised for culinary meat and that the interest in products made traditionally is greater in the United States and some European countries e.g., Hungary, Switzerland, Austria, Germany, Spain, Great Britain, and Poland. Studies [9,14] have demonstrated that good-quality pork can be obtained by crossbreeding native and commercial breeds. Such crossbreds usually show improved fattening performance and carcass composition in comparison with native breeds of pigs.

Numerous studies [8,9,20,21,22] have demonstrated that the pigs’ diet is an important environmental factor affecting pork quality, including its nutritional value. The underlying components of the diets of growing pigs are cereal grains: barley, wheat, and triticale [23,24]. Due to the presence of fibrous hulls, the use of traditional cultivars of oat (*Avena sativa* L.) in feed rations for swine is restricted. The development of naked oat cultivars (*Avena nuda* L.) containing about 2% of crude fibre is allowed use of this cereal grain in the diets of monogastric animals [25,26]. The characteristic feature of protein is a good amino acid composition with a high nutritional value. Studies by Brand and Merwe [27] demonstrated that naked oat cultivars have a higher nutritional protein value than those of other cereals, although lysine is still the limiting amino acid. A high fat level is a good source of essential unsaturated fatty acids [28,29]. A negative feature of naked oats is the presence of considerable amounts of β-glucans—antinutrients that, according to different authors [25,30,31] can account for up to 6% of dry matter. In barley, β-glucans account for 3.2–4.6% of its dry matter, in oats, 3.9–5.7%, and in wheat and triticale, 0.5–1.1% [32,33]. In order to reduce or completely eliminate the negative impact of these substances on animal bodies, it is necessary to use appropriately selected enzymatic preparations [9,34]. Numerous studies [9,21,22,27,35] have shown that complete feed rations for growing pigs could contain up to 73.5% of naked oats, but their production and slaughter performance varied. In addition, the available literature lacks studies comparing the qualification of naked oats for the diets of slow-growing breeds of pigs and their crossbreds.

Therefore, the study aimed to determine the impact of 60% naked oat on feed rations for Pulawska pigs and Pulawska x PLW (Polish Large White) crossbreds on the fattening performance, carcass composition, and quality of meat.

## 2. Materials and Methods

### 2.1. Experimental Design

The study involved 40 pigs of the Pulawska breed (P) and 40 Pulawska x PLW crossbred pigs (M). All the animals were fattened starting from average body weight of 29.5 kg up to about 102 kg. Therefore, for Pulawska x PLW crossbreds, the fattening period lasted 90 days, and for Pulawska pigs, 105 days. The pigs were kept in pens (20 animals per pen) with deep litter, equipped with automatic feed dispensers and nipple drinkers. The animals were fed *ad libitum*. Twenty Pulawska pigs and 20 Pulawska x PLW crossbreds received feed rations based on barley meal (J), and 20 Pulawska pigs and 20 Pulawska x PLW crossbreds were diets containing 60% of naked oat meal replacing 2/3 of barley (O) (Table 1).

The rations were balanced in accordance with nutritional recommendations [37], assuming the content of basic components according to the current results (Table 2) of chemical analyses in raw materials, while the remaining data were based on the tables [37]. The partial replacement of barley with naked oats increased the metabolisable energy in the experimental ration by 1.12 MJ and the lysine content by 0.9 g. However, the content of protein and lysine per 1 MJ ME was identical in both feed rations, while the percentage of extracted soybean meal in the experimental rations was reduced (O rations).

Prior to and after the experiment, all the animals were weighed individually and the results were used as a reference to calculate daily weight gains. During fattening, the feed intake (FI) was strictly controlled in order to calculate the feed conversion ratio (FCR).

### 2.2. Evaluation of the Pigs’ Carcass Composition

On the final day of the trial the pigs were slaughtered. In order to evaluate carcass composition and meat quality, 10 pigs with body weights representative of a specific group were selected from each group. According to the technology used by meat processing plants, the pigs were slaughtered two hours after transport (30 km) by electric stunning and bleeding in a horizontal position. The meatiness of the pigs was evaluated using an ULTRA-FOM 300 apparatus (SFK, Peosta, IA, USA). Afterwards, the carcasses were cooled for 24 h at a temperature of 0–4 °C. The length of the carcass was measured on the right-side half-carcass, and backfat thickness was determined at five points: above the shoulder, mid-back (between the last thoracic vertebra and the first lumbar vertebra), and over the loin (at the level of sacral vertebrae: I, II, III). Afterwards, the right-side half-carcasses were cut using the SKURTCh method [38]. During dissection, samples of *musculus longissimus lumborum* and *musculus semimembranosus* were taken from each carcass (*n* = 10 per muscle) and set up in twos (*n* = 5 per muscle) for the evaluation of the physicochemical properties of the meat.

### 2.3. Evaluation of the Chemical Composition of Naked Oats and Meat

The dry matter, total ash, crude protein, crude fat, and crude fibre contents were described using the AOAC [39] method: dry matter (930.15), total ash (942.05), crude protein (990.03), crude fat (991.36), and crude fibre (978.10) for naked oats only. The share of nitrogen-free extractives (NFE) in naked oats was calculated using the following formula:

NFE = dry matter − (crude protein + total ash + crude fat + crude fibre).

The content of amino acids in naked oats was determined by ion-exchange chromatography, using an AAA-T400 amino acid analyser made by Microtechna Prague with suitable hydrolysed proteins: tryptophan was determined by spectrophotometry using ap-dimethylaminobenzaldehyde (DAMB), methionine, and cysteine, following performic acid oxidation of the sample, and other amino acids following acid hydrolysis of protein.

Comminuted samples of oats were weighed and wet-mineralised in a mixture of HNO_3_ and HClO_4_ (at a 3:1 ratio). The mineralisation was conducted in a block made by the Tecator company with a programmed temperature. Next, in the resulting mineralisate, the contents of macroelements—calcium, sodium, and potassium—were determined by flame atomic emission spectrometry in a Flacho 4 photometer made by Carl Zeiss-Jena and that of phosphorus by colorimetry using eikonogen as a reducing agent.

The level of β-glucans in naked oats was determined via the enzymatic method of McCleary and Codd using an assay kit obtained from Megazyme International Ltd. (Bray, Ireland) [40].

Fatty acids were separated using the Folch method [41]. The fatty acid profile of the lipid fraction was determined by gas chromatography (GC) of methyl esters using a Varian 450-GC gas chromatograph with a flame ionisation detector (air–hydrogen). A Select™ Biodiesel for FAME capillary column was used (30 m, 0.32 mm, 0.25 μm) with a Select Biodiesel for FAME Fused Silica filling. The injector temperature was 250 °C, detector temperature 300 °C, and column temperature 200 °C (initial) and 240 °C (final). Helium was used as a carrier gas, with a flow of 2.5 mL per minute. The neutral or hypocholesterolemic fatty acids (DFA) and hypercholesterolemic fatty acids (OFA) were calculated based on the percentage (%) of total fatty acids [29], using the following formulas:

DFA = MUFA (monounsaturated fatty acids) + C18:0, OFA = C14:0 + C16:0.

### 2.4. Evaluation of the Physical Properties of Muscles

The concentration of hydrogen ions (pH_45_ and pH_24_) in *longissimus* muscles was measured using a Testo 205 pH meter with a dagger electrode. Forty-five minutes after the slaughter and after more than 24 h of cooling, muscle reaction (pH_45_ and pH_24_) was measured.

Water losses, expressed as water-holding capacity (WHC), were determined using Grau and Hamm’s [42] method as modified by Pohja and Ninivarra [43], based on how much free water (expressed in %) is lost by the sample of meat placed on the filter paper pressed between two glass plates. The infiltration area (cm^2^) was measured using a planimeter and calculated as the content of free water, assuming that 1 cm^2^ of filter paper absorbs 10 mg of muscle juice.

According to the L, a*, b* system, the colour of *longissimus lumborum* and *semimembranosus* muscles was determined using a Minolta Chroma Meter CR 300 (Konica Minolta, Osaka, Japan) [44]. Two illuminant/observer combinations were applied, i.e., illuminant C (average day light) and standard observer 2°, as well as illuminant D65 (day light) and standard observer 10°, recommended for measurements of meat colour [45]. In the measuring system used, L denotes psychometric colour saturation which is a spatial vector. On the other hand, a* and b* are trichromatic coordinates, where a* being a positive value corresponds to red, and being a negative value to green; in turn, positive b* corresponds to yellow and negative b* to blue. The colour parameters a* and b* were used to calculate chroma (C) and the proportion of redness to yellowness (H; hue) [46] with the following formulas:

C = [(a*)^2^ + (b*)^2^]^0.5^, H = log(b*/a*).

### 2.5. Statistical Analysis

The results were elaborated by statistical methods using a two-way analysis of variance. The significance of differences between mean values was verified at the significance level α ≤ 0.05. The obtained data were tested using the post hoc Tukey’s test. The results were processed with STATISTICA PL 13.1 software [47].

## 3. Results

A 60% inclusion of naked oats in diets had no significant (*p* > 0.05) influence on the pigs’ weight gain (Table 3). Independent from the pigs’ diet, a significantly lower (*p* ≤ 0.05) daily weight gain by about 14.5%, and higher feed conversion ratio by about 15% were recorded in Pulawska pigs compared to Pulawska x PLW crossbreds.

When analysing the carcass composition of pigs, the pigs’ diet and breed were not observed to have an impact on the pigs’ dressing percentage meatiness and backfat thickness, except that the “eye” of the loin was significantly smaller (by 9%) in Pulawska pigs compared to crossbreds of this breed with PLW pigs. No interaction effect of experimental factors (F/B) was observed in the study for the pigs’ performance results and carcass composition.

The rations fed to Pulawska pigs and Pulawska x PLW crossbreds did not affect the weights of primal cuts in the right-side half-carcass (Table 4).

Compared to Pulawska pigs, crossbreds featured a higher (*p* ≤ 0.05) weight of fillet (by 12%) and ham with shin (by 4.5%) but a significantly lower weight of bacon with ribs (by 18%). In addition, an interaction effect of both experimental factors was observed for the weight of bacon with ribs—the weight of this meat cutting was lower (*p* ≤ 0.05) in crossbred pigs receiving rations with naked oats than in other groups.

The pigs’ diet showed no impact on the evaluated muscles, except reduced (by 2.35 points) colour lightness (L) of the *musculus semimembranosus* in pigs receiving feed rations with a 60% share of naked oats in comparison to those receiving feed rations containing barley (Table 5).

The *musculus longissimus lumborum* and *musculus semimembranosus* in Pulawska pigs in comparison to crossbred pigs featured significantly improved (*p* ≤ 0.05) water-holding capacity (by 13% and 11%) and reduced colour lightness (by 1.2 and 2.6 points). In addition, the *longissimus lumborum* muscle of Pulawska pigs had a higher (by 0.97) chroma (C) and a lower (by 0.063) hue (H) in comparison to Pulawska x PLW pigs. Analysing the impact of breed and diet, an interaction (*p* ≤ 0.05) was observed for the hue of the *longissimus lumborum* muscle.

The content of essential nutrients in *musculus longissimus lumborum* and *musclus semimembranosus* did not depend on the pigs’ diet and breed, or on their interaction (F/B) except that a higher (by 18%; *p* ≤ 0.05) content of intramuscular fat was measured in the *longissimus lumborum* muscles of Pulawska pigs in comparison to those of crossbred pigs (Table 6).

Analysis of the lipid profile of intramuscular fat in pigs’ *longissimus lumborum* and *semimembranosus* muscles showed a significant impact of both experimental factors and their interactions (Table 7 and Table 8).

Naked oats included in the pigs’ diet increased (by 28% in *m. longissimus lumborum* and by 20% in *m. semimembranosus*) the share of PUFA (*p* ≤ 0.05). In addition, the muscles of Pulawska x PLW pigs contained more by 51 and 19% (*p* ≤ 0.05) polyunsaturated fatty acids (PUFA) than the muscles of the Pulawska breed in the *longissimus lumborum* and in the *semimembranosus*, respectively. The muscles of Pulawska pigs showed a significantly improved n-6/n-3 acid ratio by 66 and 69% in *m. longissimus lumborum* and *m. semimembranosus*. In the n-6/n-3 acid ratio in the *semimembranosus* muscle, an interaction effect was observed. The pigs’ diet and their breed had no significant impact on the total saturated fatty acids (SFA) and total unsaturated fatty acids (UFA) nor on neutral or hypocholesterolemic (DFA) and hypercholesterolemic fatty acids (OFA) in either of the evaluated muscles.

## 4. Discussion

A 60% inclusion of naked oats in diets for pigs had no impact (*p* > 0.05) on the daily weight gain of pigs. Meanwhile, Brand and Merwe [27], having added naked oats in amounts corresponding to 0, 24.25, 49.25, and 73.50% of pigs’ rations (25.7–89.2 kg), noted a reduction in daily weight gain by 22, 30, and 39 g, respectively, in comparison to control pigs (918 g). At the same time, grower–finisher pigs (Large White x Landrace) receiving naked oats in their feed rations showed an improved feed conversion rate. However, Morris and Burrows [22], evaluating feed rations containing 30, 65, and 96.75% of naked oats, observed an insignificant increase (by 10–20 g) in the daily weight gain of experimental pigs ((Hampshire × Duroc) × (Yorkshire × Landrace)) in comparison to the control ones (820 g). In turn, Kosieradzka and Fabijańska [48], having replaced 30% of wheat with naked oats in diets for fattening pigs, recorded a higher (872.25 g) daily weight gain than in the control group (866.25 g). Pigs receiving naked oats in feed rations showed a better feed conversion rate (2.82 kg) than the control pigs (2.96 kg). Similarly, Fabijańska and Bekta [35] demonstrated improved daily weight gain (855 g) of pigs ((Polish Large White × Polish Landrace) × (Duroc × Pietrain)) fed rations in which all barley was replaced by naked oats, with a combined feed conversion rate (2.81 kg) lower than in the pigs fed rations containing barley. Semeniuk and Grela [49] also observed a positive impact of including 50% of naked oats in cereal feed materials for fattening pigs ((Polish Landrace × Polish Large White) × Pietrain). Variability in the pigs’ weight gain and feed conversion rates can be explained by the inclusion of naked oats in their diet and by the fact that the trials involved various commercial crossbred lines.

Lower daily weight gain and a higher feed conversion rate were recorded in Pulawska pigs compared to Pulawska and PLW crossbreds in studies by Stasiak et al. [50] and POLSUS [51]. The daily weight gain of Pulawska pigs and Pulawska x PLW crossbreds throughout the fattening period should be considered good, as Stasiak et al. [50] reported a daily weight gain lower by 635 g for Pulawska and PLW crossbreds. Similarly, POLSUS [51], when evaluating the usability of pigs, mentioned a lower standard weight gain for sows and hogs of respective breeds: Pulawska: 565 and 575 g and Polish Large White (PLW): 659 and 763 g.

The present study did not show any significant impact on the pigs’ diet for their carcass composition and muscle and fat content, which corroborated the results obtained by Milczarek and Osek [25] involving crossbreds (PLW x PL) x (Duroc x Pietrain). The only exception was that the “eye” of the loin in pigs fed rations containing 60% of naked oats was about 2 cm^2^ smaller (*p* ≤ 0.05). Kosieradzka and Fabijańska [48], having replaced 30% of wheat with naked oats in the feed ration of pigs ((Polish Large White × Polish Landrace) × (Duroc x Pietrain)), noted that dressing percentage improved by 0.73% and the “eye” of the loin increased by 2.22 cm^2^. At the same time, experimental pigs featured significantly thinner backfat (25.81 mm) than those from the control group (29.07 mm). Moreover, Fabijańska et al. [21], having replaced 55% of barley with naked oats, observed that the “eye” of the loin was bigger (48.64 cm^2^) than in control pigs (46.86 cm^2^). However, the backfat of fattening pigs receiving feed rations with oats was 3.11 mm thicker when measured at five points. In turn, Brand and Merwe [27] did not find any significant differences in dressing percentage, although it increased (76.2–77.1%) as the share of naked oats was increased in the feed ration of pigs (Large White × Landrace). The authors did not record the impact of oats on the content of carcass fat. Similarly, Morris and Burrows [22] demonstrated that a higher (65 and 96.75%) share of naked oats in the pigs’ diet significantly (by about 1%) improved the dressing percentage when comparing control (77.4%) and experimental pigs (76.9%) fed rations containing 30% of naked oats. In our own studies, only the dressing percentage of pigs fed diets containing 60% of naked oats tended to increase.

Analysing carcass composition, breed was not observed to have an impact on the pigs’ dressing percentage, musculature, or fatness—only the “eye” of the loin was significantly smaller in Pulawska pigs compared to Pulawska x PLW crossbreds. In turn, Stasiak et al. [50] recorded a lower (48.1%) content of meat in the carcass and thinner (20.0 mm) backfat in Pulawska x PLW crossbreds. Improved dressing percentage (79.24%) and meatiness (55.47%), and thinner (16.9 mm) backfat at five points of measurement, were found by Kasprzyk et al. [11] in Pulawska pigs slaughtered when they reached a weight of about 100 kg. Similarly, Piórkowska et al. [12] noted thinner (19.4 mm) backfat but a bigger “eye” of the loin (48.3 cm^2^) for a lower slaughter weight. In turn, Babicz et al. [14], slaughtering Pulawska pigs weighing 135–140 kg, found a bigger “eye” of the loin (more than 58 cm^2^) and thicker backfat (nearly 41 mm).

The inclusion of 60% of naked oats in the diets of Pulawska pigs and Pulawska x PLW crossbreds had no impact on the weights of primal cuts of right-side half-carcasses, which was consistent with the results of studies carried out by Milczarek and Osek [25] involving (PLW × PL) × (Duroc × Pietrain) crossbreds.

The fact that the pigs’ diet had no impact on the physical traits of the evaluated muscles, except reduced lightness (L) of the ham muscle of pigs receiving diets with 60% of naked oats, corresponded to results obtained by Milczarek and Osek [25]. Fabijańska et al. [21] and Milczarek and Osek [25] did not note any impact of naked oats used in feed rations for commercial pigs on the water-holding capacity (WHC) of their meat.

In their studies, the *musculus longissimus lumborum* and *musculus semimembranosus* in Pulawska pigs showed significantly improved (*p* ≤ 0.05) water-holding capacity and reduced colour lightness (L) in comparison to crossbreds. According to Kasprzyk et al. [2], native breeds of pigs, including Pulawska pigs, had lower values of L in their *longissimus* muscle compared to commercial breeds. Moreover, the *longissimus lumborum* muscle of Pulawska pigs had a higher chroma (C) and a lower hue (H). Similar (6.25) pH_1_ and lower (5.59) pH_24_ values in the *longissimus* muscle of Pulawska pigs were reported by Piórkowska et al. [12] and Kasprzyk et al. [2]. Sieczkowska et al. [52] and Kasprzyk et al. [11] claimed that the acidification of the muscle tissue at a post mortem time of 24 h is a very important and significant parameter for meat processing plants. The reported reduction in pH at a post mortem time of 24 h is an indicator of normal meat maturation. The reported pH_24_ values for all the genetic groups were typical of normal meat [53].

The content of essential nutrients in *musculus longissimus lumborum* and *musclus semimembranosus* did not depend on the diet and breed of pigs, except that a higher (*p* ≤ 0.05) content of intramuscular fat was found in the *longissimus lumborum* muscle of Pulawska pigs. The lack of impact of a 60% inclusion of naked oats in feed rations for pigs on the content of essential nutrients in the *longissimus* muscle was concurrent with the results of Fabijańska and Bekta [35] and Milczarek and Osek [25]; however, they carried out their studies on commercial (fast-growing) pigs. Florowski et al. [54], comparing the essential nutrient compositions of the *longissimus* muscle in Pulawska pigs and Polish Landrace (PL) pigs noted a significantly higher dry matter, protein, and intramuscular fat content. In turn, Kasprzyk and Bougnacka [4] showed similar contents (2.78% vs. 2.50 and 2.51%) of fat in the *longissimus lumborum* muscle of Pulawska pigs compared to DanBred and Naima breeds. Intramuscular fat content is an important indicator of meat quality and its qualification for culinary purposes. According to Wajda et al. [55], Łyczyński et al. [56], and Grześkowiak et al. [57], the optimum level of this component in the *longissimus* muscle should be 2–3%. Intramuscular fat (IMF) content in the muscle measured in our own study was relatively low, but similar to that noted in the muscle of Pulawska pigs by Kasprzyk et al. [11] and Piórkowska et al. [12]. A lower (≤ 1%) content of intramuscular fat could deteriorate the flavour of the meat which, particularly after heat treatment, becomes dry and stringy [58]; on the other hand, a higher content of IMF (above 3.5%) can contribute to lower evaluations of meat by consumers due to the visible fat deposits [59].

The composition and share of fatty acids in the lipid fraction of muscles testifies to the dietary value of meat [60,61]. A positive impact of including naked oats in the feed ration was observed on the fatty acid profile of IMF. A significant increase in the share of PUFA, and particular linoleic acid (C18:2), classified as a so-called essential fatty acid (EFA), was observed in both muscles. These data corroborate the results of previous studies by Milczarek and Osek [25], which also showed increased levels of PUFA in the meat of commercial fattening pigs ((PLW × PL) × (Duroc × Pietrain)) fed rations containing naked oats.

The pigs’ diet and their breed showed no significant impact on the total saturated fatty acids (SFA) and total unsaturated fatty acids (UFA), nor on neutral or hypocholesterolemic (DFA) and hypercholesterolemic fatty acids (OFA) in either of the evaluated muscles. The muscles of Pulawska x PLW pigs were found to contain more (*p* ≤ 0.05) polyunsaturated fatty acids (PUFA), but the muscles of Pulawska pigs featured a significantly healthier n-6/n-3 ratio. Less SFA, but more PUFA, and simultaneously a lower share of PUFA n-3 in the muscles of Pulawska pigs, were measured by Cebulska et al. [62] and Milczarek et al. [3], which can testify to the positive impact of the pigs’ diet, and in particular the inclusion of naked oats. Migdał et al. [63] claim that slow-growing pigs show a lipid fraction with an improved fatty acid profile. However, a higher concentration of polyunsaturated fatty acids in intramuscular fat or fat reserves has an adverse effect on the technological and sensory traits of the resulting meat product.

## 5. Conclusions

To sum up, a 60% inclusion of naked oats in the diets of Pulawska pigs and Pulawska x PLW crossbreds resulted in a similar daily weight gain at a slightly higher feed conversion rate compared to pigs fed rations containing barley as the only grain. The diet had no impact on the carcass composition (dressing percentage, fatness, and musculature), essential nutrient contents, or physical traits of the muscles; however, naked oats in pigs’ diets significantly increased the share of PUFA in both muscles. Pulawska pigs showed worse fattening and at-slaughter results but their meat was of good quality, with improved water-holding capacity and nutritive value measured as the n-6/n-3 acid ratio.

## Figures and Tables

**Table 1:** Raw materials (%) and nutritive value of diets.

Item	Diet	
	J	O
Raw materials		
Barley	81.7	26.7
Naked oats	-	60
Extracted soybean meal	15.5	10.5
1-Ca phosphate	0.300	0.300
Premix *	2.50	2.50
Calculated nutritive value per 1 kg of rations		
Metabolisable energy ** (MJ)	12.29	13.32
Crude protein (g)	162	176
Lys (g)	9.05	9.95
Met + cys (g)	5.78	6.68
Thr (g)	5.84	5.57
Trp (g)	1.86	1.99
Ca (g)	6.78	6.86
P (g)	5.29	5.20
Na (g)	1.39	1.55

J—rations with barley (the only cereal grain), O—rations with naked oats. * Premix contained: metabolisable energy—min. 1.25 MJ/kg, crude protein—min. 90 g/kg, lys—min. 65 g/kg, met + cys—min. 7 g/kg, thr—min. 12.5 g/kg, Ca—min. 220 g/kg, P available—min. 43 g/kg, Na—min. 53 g/kg. ** Metabolisable energy was calculated according to the equation proposed by Kirchgessner and Roth [36].

**Table 2:** Chemical composition of naked oats.

Item	Contents ± SD
Essential nutrients (g/kg dry matter)	
Dry matter	885 ± 13.2
Crude ash	23.73 ± 0.174
Crude protein	186.44 ± 2.53
Crude fat	57.28 ± 1.07
Crude fibre	29.72 ± 1.37
Nitrogen-free extractives (NFE)	587.83 ± 16.1
Amino acids (g/16 g N)	
Lys	4.42 ± 0.140
Met	1.43 ± 0.089
Cys	2.55 ± 0.110
Thr	3.76 ± 0.117
Trp	1.02 ± 0.104
Macroelements (g/kg)	
Ca	0.620 ± 0.020
P	5.11 ± 0.110
K	3.09 ± 0.101
Na	0.108 ± 0.013
β-glucans (% dry matter)	4.28 ± 0.295

SD—standard deviation, *n* = 3.

**Table 3:** Fattening performance and carcass composition.

Item	Breed (B)	Diet (F)		$\bar{x}$	*p*-Value			SEM
		J	O		F	B	F/B	
Body weight (kg) - initial	P	29.8	29.7	29.75	0.501	0.436	0.492	0.975
	M	29.6	29.2	29.40				
	$\bar{x}$	29.70	29.45					
- final	P	102.7	102.1	102.40	0.534	0.582	0.615	0.888
	M	102.3	102.3	102.30				
	$\bar{x}$	102.50	102.20					
Daily weight gain (g)	P	694	690	692 ^b^	0.724	<0.05	0.053	0.682
	M	808	813	810 ^a^				
	$\bar{x}$	751	751					
FCR (kg)	P	3.10	3.24	3.17	-	-	-	-
	M	2.74	2.77	2.76				
	$\bar{x}$	2.92	3.01					
Dressing percentage (%)	P	76.3	77.4	76.9	0.343	0.681	0.058	0.290
	M	76.9	77.1	77.0				
	$\bar{x}$	76.6	77.3					
Meatiness (%)	P	51.2	50.0	50.6	0.597	0.049	0.221	0.467
	M	53.0	53.0	53.0				
	$\bar{x}$	52.1	51.5					
Loin “eye” area (cm^2^)	P	45.5	45.0	45.3 ^b^	0.856	<0.05	0.968	1.07
	M	50.0	49.6	49.8 ^a^				
	$\bar{x}$	47.8	47.3					
Lard weight (kg)	P	1.68	1.76	1.72	0.323	0.134	0.319	0.063
	M	1.55	1.44	1.50				
	$\bar{x}$	1.62	1.60					
Carcass length (cm)	P	80.4	79.8	80.1	0.901	0.483	0.205	0.297
	M	80.5	80.4	80.5				
	$\bar{x}$	80.5	80.1					
Backfat thickness (mm) - over the shoulder	P	37.33	42.7	40.02	0.305	0.163	0.156	1.00
	M	37.43	37.1	37.27				
	$\bar{x}$	37.38	39.90					
- mid back	P	19.78	25.6	22.69	0.053	0.873	0.055	0.692
	M	22.78	23.00	22.89				
	$\bar{x}$	21.28	24.30					
- sacrum I	P	28.11	29.50	28.81	0.932	0.126	0.566	0.901
	M	26.51	25.20	25.86				
	$\bar{x}$	27.31	27.35					
- sacrum II	P	18.78	22.00	20.39	0.275	0.678	0.753	0.876
	M	18.58	19.2	18.89				
	$\bar{x}$	18.68	20.60					
- sacrum III	P	31.00	34.10	32.55	0.955	0.103	0.291	1.35
	M	29.60	26.10	27.85				
	$\bar{x}$	30.30	30.10					
- arithmetic mean from 5 measurements	P	27.00	30.36	28.68	0.448	0.179	0.189	0.818
	M	26.98	26.12	26.55				
	$\bar{x}$	26.99	28.24					

J—rations with barley (the only cereal grain), O—rations with naked oats, FCR—feed conversion ratio, B—breed, F—diet, SEM—standard error of the mean, *n* = 20 for fattening performance, *n* = 10 for carcass composition traits, ab—means with different superscripts are significantly different at *p* ≤ 0.05.

**Table 4:** Primal cuts weight (kg) of right half-carcass.

Item	Breed (B)	Diet (F)		$\bar{x}$	*p*-Value			SEM
		J	O		F	B	F/B	
neck	P	5.84	6.20	6.02	0.351	0.077	0.499	0.114
	M	6.53	6.42	6.48				
	$\bar{x}$	6.19	6.31					
shoulder	P	4.32	4.15	4.24	0.052	0.065	0.078	0.151
	M	3.98	3.90	3.94				
	$\bar{x}$	4.15	4.03					
bacon and ribs	P	6.31 ^a^	6.53 ^a^	6.42 ^a^	0.063	<0.05	<0.05	0.154
	M	5.75 ^a^	4.75 ^b^	5.25 ^b^				
	$\bar{x}$	6.03	5.64					
fillet	P	8.24	7.75	8.00 ^b^	0.053	<0.05	0.490	0.153
	M	8.66	9.34	9.00 ^a^				
	$\bar{x}$	8.45	8.55					
− longissimus	P	2.61	2.77	2.69	0.591	0.053	0.058	0.060
	M	2.82	3.02	2.92				
	$\bar{x}$	2.72	2.90					
− small meat	P	1.56	1.47	1.52 ^b^	0.051	<0.05	0.097	0.506
	M	1.86	2.37	2.12 ^a^				
	$\bar{x}$	1.71	1.92					
− bones	P	1.14	1.16	1.15 ^b^	0.133	<0.05	0.192	0.022
	M	1.24	1.32	1.28 ^a^				
	$\bar{x}$	1.19	1.24					
− skin + fat	P	2.93	2.35	2.64	0.320	0.682	0.160	0.105
	M	2.69	2.58	2.64				
	$\bar{x}$	2.81	2.47					
ham with shin	P	9.58	9.79	9.69 ^b^	0.377	<0.05	0.852	0.103
	M	10.2	10.03	10.12 ^a^				
	$\bar{x}$	9.89	9.91					
− ham	P	8.59	8.86	8.73	0.201	0.129	0.812	0.102
	M	9.19	8.97	9.08				
	$\bar{x}$	8.89	8.92					
meat	P	6.28	6.68	6.48 ^b^	0.372	<0.05	0.626	0.104
	M	6.78	6.23	6.51 ^a^				
	$\bar{x}$	6.53	6.46					
bones	P	0.61	0.62	0.62	0.520	0.054	0.146	0.009
	M	0.66	0.66	0.66				
	$\bar{x}$	0.64	0.64					
skin + fat	P	1.7	1.56	1.63	0.877	0.581	0.383	0.052
	M	1.62	1.56	1.59				
	$\bar{x}$	1.66	1.56					
− shin	P	0.99	0.93	0.96	0.106	0.066	0.152	0.011
	M	1.02	1.06	1.04				
	$\bar{x}$	1.01	1.00					

J—rations with barley (the only cereal grain), O—rations with naked oats, B—breed, F—diet, SEM—standard error of the mean, *n* = 10, ab—means with different superscripts are significantly different at *p* ≤ 0.05.

**Table 5:** Physical properties of meat.

Item	Breed (B)	Diet (F)		$\bar{x}$	*p*-Value			SEM
		J	O		F	B	F/B	
*Longissimus lumborum*								
pH _45_	P	6.23	6.36	6.30	0.069	0.347	0.902	0.039
	M	6.15	6.28	6.22				
	$\bar{x}$	6.19	6.32					
pH _24_	P	5.62	5.58	5.60	0.101	0.288	0.879	0.035
	M	5.65	5.54	5.60				
	$\bar{x}$	5.64	5.56					
WHC (%)	P	19.6	21.3	20.4 ^b^	0.148	<0.05	0.813	0.699
	M	22.5	24.7	23.6 ^a^				
	$\bar{x}$	21.1	23.0					
Colour								
L*	P	51.3	51.2	51.2	0.986	0.929	0.279	0.457
	M	52.2	52.6	52.4				
	$\bar{x}$	51.7	51.9					
a*	P	9.18	8.16	8.67 ^a^	0.109	<0.05	0.114	0.180
	M	7.63	7.62	7.63 ^b^				
	$\bar{x}$	8.41	7.89					
b*	P	0.768	0.757	0.763	0.105	0.066	0.119	0.115
	M	1.51	0.818	1.164				
	$\bar{x}$	1.139	0.788					
C = [(a*)^2^ + (b*)^2^]^0.5^	P	9.23	8.21	8.72 ^a^	0.072	<0.05	0.172	0.179
	M	7.82	7.68	7.75 ^b^				
	$\bar{x}$	8.53	7.95					
H = log(b*/a*)	P	0.081 ^b^	0.092 ^b^	0.087 ^b^	0.093	<0.05	<0.05	0.015
	M	0.200 ^a^	0.101 ^b^	0.151 ^a^				
	$\bar{x}$	0.141	0.097					
*Semimembranosus*								
WHC (%)	P	19.7	17.5	18.6 ^b^	0.225	<0.05	0.102	0.429
	M	21.1	20.8	20.9 ^a^				
	$\bar{x}$	20.4	19.1					
Colour								
L*	P	45.9	44.6	45.2 ^b^	<0.05	<0.05	0.288	0.556
	M	49.5	46.1	47.8 ^a^				
	$\bar{x}$	47.7 ^a^	45.4 ^b^					
a*	P	11.4	11.9	11.7	0.107	0.215	0.724	0.199
	M	10.8	11.6	11.2				
	$\bar{x}$	11.1	11.8					
b*	P	1.34	0.919	1.130	0.562	0.415	0.500	0.163
	M	0.838	0.879	0.858				
	$\bar{x}$	1.089	0.899					
C = [(a*)^2^ + (b*)^2^]^0.5^	P	11.6	12.0	11.8	0.153	0.174	0.676	0.194
	M	10.9	11.6	11.3				
	$\bar{x}$	11.3	11.8					
H = log(b*/a*)	P	0.121	0.071	0.096	0.358	0.592	0.601	0.015
	M	0.091	0.071	0.081				
	$\bar{x}$	0.106	0.071					

J—rations with barley (the only cereal grain), O—rations with naked oats, B—breed, F—diet, SEM——standard error of the mean, *n* = 10, ab—means with different superscripts are significantly different at *p* ≤ 0.05, L*—lightness, a*—redness, b*—yellowness, C—chroma, H—hue, WHC—water-holding capacity.

**Table 6:** Chemical composition (g/100 g) of meat.

Item	Breed (B)	Diet (F)		$\bar{x}$	*p*-Value			SEM
		J	O		F	B	F/B	
*Longissimus lumborum*								
Dry matter	P	25.54	25.98	25.76	0.266	0.105	0.105	0.140
	M	25.89	25.63	25.76				
	$\bar{x}$	25.71	25.80					
Crude ash	P	1.13	1.11	1.12	0.129	0.194	0.059	0.005
	M	1.11	1.16	1.13				
	$\bar{x}$	1.12	1.13					
Crude protein	P	22.65	22.79	22.72	0.391	0.106	0.095	0.840
	M	22.66	22.25	22.46				
	$\bar{x}$	22.66	22.52					
Crude fat	P	1.75	2.08	1.91 ^a^	0.716	<0.05	0.183	0.114
	M	2.01	1.22	1.61 ^b^				
	$\bar{x}$	1.88	1.65					
*Semimembranosus*								
Dry matter	P	24.69	24.86	24.78	0.800	0.256	0439	0.134
	M	25.00	24.60	24.80				
	$\bar{x}$	24.85	24.73					
Crude ash	P	1.10	1.09	1.10	0.845	0.559	0131	0.005
	M	1.09	1.11	1.10				
	$\bar{x}$	1.10	1.10					
Crude protein	P	22.04	21.97	22.01	0.770	0.660	0.906	0.073
	M	21.95	21.92	21.94				
	$\bar{x}$	22.00	21.95					
Crude fat	P	1.53	1.76	1.65	0.885	0.161	0.170	0.091
	M	1.93	1.25	1.59				
	$\bar{x}$	1.73	1.51					

J—rations with barley (the only cereal grain), O—rations with naked oats, B—breed, F—diet, SEM—standard error of the mean, *n* = 5. ab—means with different superscripts are significantly different at *p* ≤ 0.05.

**Table 7:** Fatty acid profile (% total FA) of *longissimus lumborum* meat.

Item	Breed (B)	Diet (F)		$\bar{x}$	*p*-Value			SEM
		J	O		F	B	F/B	
C 14:0	P	1.01	1.00	1.01	0.082	0.132	0.183	0.017
	M	1.01	0.91	0.96				
	$\bar{x}$	1.01	0.96					
C 16:0	P	27.93 ^ab^	28.50 ^a^	28.22	0.206	0.179	<0.05	0.265
	M	28.46 ^a^	26.70 ^b^	27.58				
	$\bar{x}$	28.20	27.60					
C 18:0	P	10.37 ^b^	10.90 ^a^	10.64	0.980	0.652	<0.05	0.171
	M	10.96 ^a^	10.21 ^b^	10.59				
	$\bar{x}$	10.67	10.56					
C 18:1 n-9	P	52.63 ^a^	51.32 ^ab^	51.98	0.948	0.157	<0.05	0.267
	M	50.52 ^b^	51.98 ^ab^	51.25				
	$\bar{x}$	51.58	51.65					
C 18:2 n-6	P	2.68	3.39	3.04 ^b^	<0.05	<0.05	0.497	0.283
	M	4.08	5.32	4.70 ^a^				
	$\bar{x}$	3.38 ^b^	4.36 ^a^					
C 18:3 n-3	P	0.07	0.11	0.09	<0.05	0.489	0.611	0.011
	M	0.07	0.14	0.11				
	$\bar{x}$	0.07 ^b^	0.13 ^a^					
C 20:0	P	0.07	0.06	0.07	0.205	0.474	0.665	0.003
	M	0.06	0.05	0.06				
	$\bar{x}$	0.07	0.06					
C 20:1 n-9	P	0.33	0.33	0.33	0.646	0.230	0.646	0.012
	M	0.31	0.28	0.30				
	$\bar{x}$	0.32	0.31					
C 20:2 n-6	P	0.06	0.06	0.06	0.449	0.240	0.333	0.004
	M	0.06	0.08	0.07				
	$\bar{x}$	0.06	0.07					
C 20:3 n-3	P	0.03 ^a^	0.02 ^b^	0.03	0.600	0.600	<0.05	0.001
	M	0.02 ^b^	0.03 ^a^	0.03				
	$\bar{x}$	0.03	0.03					
C 20:4 n-6	P	0.21 ^ab^	0.18 ^b^	0.20 ^b^	0.233	<0.05	<0.05	0.018
	M	0.22 ^ab^	0.32 ^a^	0.27 ^a^				
	$\bar{x}$	0.22	0.25					
SFA	P	39.44 ^ab^	40.70 ^a^	40.07	0.321	0.218	<0.05	0.395
	M	40.53 ^a^	37.92 ^b^	39.23				
	$\bar{x}$	39.99	39.31					
UFA	P	60.39 ^ab^	59.40 ^b^	59.90	0.301	0.250	<0.05	0.389
	M	59.28 ^b^	61.87 ^a^	60.58				
	$\bar{x}$	59.84	60.64					
MUFA	P	57.35 ^a^	55.65 ^b^	56.50 ^a^	0.418	<0.05	<0.05	0.296
	M	54.83 ^b^	55.99 ^b^	55.41 ^b^				
	$\bar{x}$	56.09	55.82					
PUFA	P	3.04	3.75	3.40 ^b^	<0.05	<0.05	0.404	0.308
	M	4.45 ^b^	5.88 ^a^	5.17 ^a^				
	$\bar{x}$	3.75	4.82					
DFA	P	70.76 ^ab^	70.50 ^b^	70.63	0.151	0.187	<0.05	0.270
	M	70.24 ^b^	72.08 ^a^	71.16				
	$\bar{x}$	70.50	71.29					
OFA	P	28.94 ^ab^	29.50 ^a^	29.22	0.182	0.162	<0.05	0.276
	M	29.46 ^a^	27.61 ^b^	28.54				
	$\bar{x}$	29.20	28.56					
n-6/n-3	P	29.5	29.3	29.40 ^b^	0.570	<0.05	0.596	3.00
	M	47.4	41.5	44.45 ^a^				
	$\bar{x}$	38.45	35.40					

J—rations with barley (the only cereal grain), O—rations with naked oats, B—breed, F—diet, SEM—standard error of the mean, *n* = 5, ab—means with different superscripts are significantly different at *p* ≤ 0.05, SFA—saturated fatty acids, UFA—unsaturated fatty acids, MUFA—monounsaturated fatty acids, PUFA—polyunsaturated fatty acids, DFA—neutral or hypocholesterolemic fatty acids = MUFA + C18:0, OFA—hypercholesterolemic fatty acids = C14:0 + C16:0.

**Table 8:** Fatty acid profiles (% total FA) of *semimembranosus* meat.

Item	Breed (B)	Diet (F)		$\bar{x}$	*p*-Value			SEM
		J	O		F	B	F/B	
C 14:0	P	0.92	0.92	0.92 ^a^	0.570	<0.05	0.523	0.015
	M	0.87	0.83	0.85 ^b^				
	$\bar{x}$	0.90	0.88					
C 16:0	P	25.51	25.32	25.42	0.681	0.829	0.634	0.098
	M	25.45	25.47	25.46				
	$\bar{x}$	25.48	25.40					
C 18:0	P	9.7	9.27	9.49	0.154	0.971	0.644	0.108
	M	9.6	9.38	9.49				
	$\bar{x}$	9.65	9.33					
C 18:1 n-9	P	53.14	53.03	53.09	0.466	0.365	0.633	0.232
	M	52.91	52.33	52.62				
	$\bar{x}$	53.03	52.68					
C 18:2 n-6	P	4.66	5.79	5.23 ^b^	<0.05	<0.05	0.847	0.240
	M	5.6	6.86	6.23 ^a^				
	$\bar{x}$	5.13 ^b^	6.33 ^a^					
C 18:3 n-3	P	0.09	0.15	0.12	0.123	0.057	0.051	0.008
	M	0.1	0.09	0.10				
	$\bar{x}$	0.10	0.12					
C 20:0	P	0.06 ^a^	0.02 ^b^	0.04 ^b^	<0.05	<0.05	<0.05	0.003
	M	0.06 ^a^	0.05 ^a^	0.06 ^a^				
	$\bar{x}$	0.06 ^a^	0.04 ^b^					
C 20:1 n-9	P	0.32	0.3	0.31	0.109	0.065	0.677	0.007
	M	0.30	0.27	0.29				
	$\bar{x}$	0.31	0.29					
C 20:2 n-6	P	0.09 ^a^	0.06 ^b^	0.08 ^b^	0.115	<0.05	<0.05	0.005
	M	0.10 ^a^	0.11 ^a^	0.11 ^a^				
	$\bar{x}$	0.10	0.09					
C 20:3 n-3	P	0.04	0.04	0.04	0.569	0.346	0.703	0.004
	M	0.04	0.05	0.05				
	$\bar{x}$	0.04	0.05					
C 20:4 n-6	P	0.47	0.39	0.43 ^b^	0.758	<0.05	0.117	0.035
	M	0.51	0.63	0.57 ^a^				
	$\bar{x}$	0.49	0.51					
SFA	P	36.23	35.58	35.91	0.262	0.986	0.621	0.190
	M	36.03	35.77	35.90				
	$\bar{x}$	36.13	35.68					
UFA	P	63.54	64.17	63.86	0.277	0.956	0.624	0.188
	M	63.75	64	63.88				
	$\bar{x}$	63.65	64.09					
MUFA	P	58.17	57.75	57.96	0.173	0.053	0.521	0.298
	M	57.39	56.26	56.83				
	$\bar{x}$	57.78	57.01					
PUFA	P	5.36	6.42	5.89 ^b^	<0.05	<0.05	0.676	0.260
	M	6.36	7.74	7.05 ^a^				
	$\bar{x}$	5.86 ^b^	7.08 ^a^					
DFA	P	73.24	73.44	73.34	0.622	0.894	0.685	0.103
	M	73.36	73.38	73.37				
	$\bar{x}$	73.30	73.41					
OFA	P	26.43	26.24	26.34	0.649	0.923	0.721	0.104
	M	26.32	26.3	26.31				
	$\bar{x}$	26.38	26.27					
n-6/n-3	P	37.1 ^b^	35.0 ^b^	36.05 ^b^	0.062	<0.05	<0.05	2.29
	M	45.6 ^b^	58.2 ^a^	51.90 ^a^				
	$\bar{x}$	38.65	44.10					

J—rations with barley (the only cereal grain), O—rations with naked oats, B—breed, F—diet, SEM—standard error of the mean, *n* = 5, ab—means with different superscripts are significantly different at *p* ≤ 0.05, SFA—saturated fatty acids, UFA—unsaturated fatty acids, MUFA—monounsaturated fatty acids, PUFA—polyunsaturated fatty acids, DFA—neutral or hypocholesterolemic fatty acids = MUFA + C18:0, OFA—hypercholesterolemic fatty acids = C14:0 + C16:0.

## Data Availability

Data is available upon request from the corresponding author.
